# *In silico* identification and characterization of novel microsatellite loci for the Blue-and-yellow Macaw *Araararauna* (Linnaeus, 1758) (Psittaciformes, Psittacidae)

**DOI:** 10.1590/1678-4685-GMB-2017-0338

**Published:** 2019-01-31

**Authors:** Cássia Alves Lima-Rezende, Gislaine Aparecida Fernandes, Helder Elias da Silva, Sarah Dobkowski-Marinho, Victor Fernandes Santos, Fernando Pacheco Rodrigues, Renato Caparroz

**Affiliations:** 1 Universidade de Brasília Universidade de Brasília Instituto de Ciências Biológicas Departamento de Genética e Morfologia Asa Norte, BrasiliaDF Brazil Laboratório de Genética e Biodiversidade, Departamento de Genética e Morfologia, Instituto de Ciências Biológicas, Universidade de Brasília, Campus Universitário Darcy Ribeiro, Asa Norte, Brasilia, DF, Brazil

**Keywords:** Captive breeding, illegal wildlife trade, parentage, Psittacidae, wildlife forensics

## Abstract

The illegal trade is a major threat to many bird species, and parrots are common victims of this activity. Domestic and international pet markets are interested on different parrot species, such as the Blue-and-yellow Macaw (*Ara ararauna*). This South American macaw is not globally threatened, but is under protection from over-exploitation. This study aimed to identify and characterize novel microsatellite loci for population and parentage analysis of *A. ararauna*. Scaffold sequences of *Ara macao* available in the NCBI database were used for microsatellite searches using MsatCommander software. We tested a total of 28 loci, from which 25 were polymorphic, one was monomorphic, and two did not generated amplification products. For polymorphic loci, the mean number of alleles was 8.24 (4 – 15 alleles per locus), the observed heterozygosity ranged from 0.333 to 0.917, and the expected heterozygosity from 0.353 to 0.890. The paternity exclusion probability and identity probability were highly discriminatory. Thus, these novel microsatellite markers can be useful for population assignment and paternity tests, helping the authorities to manage macaws from the illegal trafficking and control commercial breeders.

The illegal trade of plants and animals, as well as their parts and products, is a diverse and profitable business. This business involves billions of dollars, and millions of animals and plants are caught or harvested from the wild and then sold every year around the world (Webb, 2001; Broad *et al.*, 2003). Brazil is one of the major suppliers of the international wildlife trade, and it is estimated that birds represent almost 80% of the Brazilian market of exotic animals (RENCTAS, 2001; Broad *et al.*, 2003). For instance, the Lear’s Macaw (*Anodorhynchus leari*), Hyacinth Macaw (*Anodorhynchus hyacinthinus*), Blue-and-yellow Macaw (*Ara ararauna*), and Red-tailed Amazon (*Amazona brasiliensis*) are Brazilian parrots commonly sold to illegal pet collectors of Asia, Europe, and North America (RENCTAS, 2001).

Almost half of the 176 parrot species in the Neotropics are threatened to some degree of extinction (Olah *et al.*, 2016). The main threats to Neotropical parrots are the habitat loss due to the expansion of agricultural activities, hunting and trapping wild animals for the domestic and international pet markets, and logging (Olah *et al.*, 2016; Berkusky *et al.*, 2017). Every year, thousands of parrots are removed from their natural habitat, and the direct and indirect effects of the illegal trade have led to a reduction of some natural populations (Pires, 2012; Berkusky *et al.*, 2017). In general, nestlings illegally removed from their natural habitat are directly sold in fairs and on the margins of Brazilian roads, while others are “laundered” by some legal Brazilian commercial breeders and sold in pet shops as born in captivity.

Although a great number of birds have been confiscated from traffickers and illegal owners, the geographic origin of these animals is frequently unknown, making it very difficult to establish an appropriate wild site where to return these birds (IUCN, 2002). The indiscriminate release of confiscated animals may have negative genetic impacts on local populations, since offspring produced by crosses between individuals from different subpopulations may show signs of reduced fitness (Marshall and Spalton, 2000), known as outbreeding depression. Additionally, the parentage verification is needed to provide evidence of illegal activity of a suspicious commercial breeder (Jan and Fumagalli, 2016).

In this sense, molecular markers, such as microsatellites, constitute a useful tool for population genetics (Selkoe and Toonen, 2006) and forensic studies (Miller and Bloomer, 2014). In forensics studies of illegal wildlife trade, authorities may use microsatellites in different ways, such as (1) in DNA-based parentage tests to control commercial breeders, avoiding the laundering of wild-caught individuals as captive-bred (Jan and Fumagalli, 2016); and (2) to help in the identification of the probable geographical origin of seized animals by assignment tests (Presti *et al.*, 2015). Although microsatellites have been the preferential markers in forensics studies of non-model species, a relatively limited number of loci is available for parrots (Presti and Wasko, 2014), and only few were isolated for *A. ararauna* (Caparroz *et al.*, 2003).

Here, our goal was to identify and characterize novel polymorphic microsatellite loci for the Blue-and-yellow Macaw (*A. ararauna*) based on the previously published draft-genome of a congeneric species (*Ara macao*). The Blue-and-yellow Macaw is a South American species from the Psittacidae family, which is not globally threatened, but is under protection from over-exploitation (CITES II) (Collar *et al.*, 2017). It is estimated that from 1981–1992 approximately 42 thousand individuals of *A. ararauna* were traded internationally (Broad *et al.*, 2003). The set of microsatellite loci described in this study can be useful for population assignment and parentage analysis, helping the authorities to manage victims of illegal trafficking and to control commercial breeders.

The scaffold assembly of *Ara macao* (Seabury *et al.*, 2013), publicly available under the accession AOUJ00000000.1 in the NCBI database, was used for genome-wide *in silico* microsatellites searches. Scaffolds were screened for tri-, tetra-, penta-, and hexa-nucleotide microsatellite motifs with 10 or more tandem repeats using the software MSATCOMMANDER 1.0.8 (Faircloth, 2008). The search for locus-specific primers was done using PRIMER3Web (Untergasser *et al.*, 2012) and considering PCR products within a size range between 170 and 350 bp, optimal primers length of 20 bp, optimal melting temperature of 58 °C (56 °C – 60 °C), GC content between 40 and 60%, and other parameters set as default. One primer per locus was tagged using an M13 universal sequence (5’–TGT AAA ACG ACG GCC AGT–3’) on its 5’ end to apply the dye-labeling method proposed by Schuelke (2000). Finally, the online software OLIGO-ANALYZER 3.1 (Integrated DNA Technologies, Coralville, IA) was used to estimate the occurrence of hairpins, self-dimers, and heterodimers among primers.

Total DNA from 24 *A. ararauna* blood samples was isolated using proteinase K digestion followed by phenol-chloroform extraction (Bruford *et al.*, 1992). As the *A. macao* scaffold assembly was used for microsatellite searching, we also extracted the total DNA from one *A. macao* blood sample for use as a positive control in the PCR assays. *A. ararauna* samples were collected in three different localities in Brazil: 14 from Alto Paraíso de Goiás (14°03’49.0"S; 47°39’48.4"W), 15 from Parque Nacional das Emas (18°09’14.4"S; 52°51’09.4"W), and five from Corguinho (19°54’20.3"S; 54°49’46"W). The *A. macao* sample was collected from an individual kept in captivity and of unknown origin.

Each locus was initially amplified in eight *A. ararauna* specimens, and the loci that showed success in amplification and polymorphism were used to genotype the other 16 sampled individuals. PCR assays were performed in a 10 μL volume containing 20–40 ng of template DNA, 1X PCR buffer, 0.25 mM of each dNTP, 0.08 mM of primer with M13 tail, 0.32 mM of untailed primer, 0.32 mM of fluorescent M13 primer (FAM, HEX, or NED fluorophores, Applied Biosystems, CA), 1.5 mM MgCl_2_, and 0.5 unit of *Taq* polymerase (Invitrogen). Thermocycling conditions were as follows: 94 °C for 7 min, then 10 cycles at 94 °C 1 min, the locus-specific annealing temperature for 1 min (see Table 1), 72 °C for 45 s, followed by 30 cycles at 94 °C for 1 min, 53 °C for 1 min, 72 °C for 45 s, and a final extension step at 72 °C for 10 min. Negative (Milli-Q water) and positive (*A. macao* DNA sample) controls were included in each reaction set. Amplification success was confirmed by electrophoresis on 1% agarose gel stained with ethidium bromide. A multiplex reaction containing 0.7 μL of PCR product of three different loci (each one labeled with a different fluorescent dye) was added to 7.4 μL of formamide and 0.5 μL of GeneScan 500 ROX size standard (Applied Biosystems), denatured at 95 °C for 5 min and separated by capillary electrophoresis in an ABI PRISM 3130 DNA sequencer (Applied Biosystems). Allele sizes were scored in GENEIOUS 6.1.6 software using the Microsatellites Plugin (Biomatters). To confirm the presence of the respective microsatellite locus, one individual (preferably homozygous) for each locus was amplified using the same PCR conditions described above, but using a non-fluorescent M13 primer. We used the Shrimp Alkaline Phosphatase and Exonuclease I protocol to clean the PCR products, and the sequencing was performed by Macrogen (Seoul, Korea).

**Table 1:** Heterologous primer sequences and characteristics of 28 microsatellite loci for the Blue-and-yellow Macaw (*Ara ararauna*) drawn from the *Ara macao* genome. Annealing temperature (Ta) is given in degrees Celsius.

Locus	NCBI Accession	Primer (5’–3’)	Motif	Ta (°C)
Amac-01	AOUJ01027422.1	F: ACCAACCAAACATGAACAGA	ATTCT_(14)_	54
		R: CCTTCCTCACGGTTCTTACT		
Amac-02	AOUJ01101673.1	F: CCTTCTTGTTGATCTGCTTG	GGCAA_(13)_	54
		R: TCTGTCATTAGGAGGCTGAA		
Amac-03	AOUJ01106782.1	F: AGAATGAGTCCCAGGCTTAC	CCACA_(13)_	56
		R: ACAAGGCAGATAGCACAAGA		
Amac-04	AOUJ01379433.1	F: CCTGAACCAATGTGCTCTAC	CCAT_(30)_	54
		R: GACAGGGAACTGGGACAGAT		
Amac-05	AOUJ01197926.1	F: TGCAGCTAGTTTGGTCTTGT	AGGA_(24)_	54
		R: AGTTACCTAAAGCCTGCCTG		
Amac-06	AOUJ01268066.1	F: AGGGACATGCAGGAAAGTAT	AGAT_(15)_	54
		R: TTAAGTTCCAGGGGGAAGTA		
Amac-07	AOUJ01078670.1	F: GTCCAAATCCATCTGTTTCA	TCTA_(15)_	56
		R: CCTTTAGCCTCCTCTCACAT		
Amac-08	AOUJ01152411.1	F: CGAGAAGTTTGAAGTTGCAG	ATCT_(15)_	54
		R: CCAAGCACTATCTTCCCTCT		
Amac-09	AOUJ01055174.1	F: TCAAACCCAAATCACTGTTC	ATCT_(15)_	54
		R: AAGAAGTGGTGTTCCCTGAT		
Amac-10	AOUJ01141428.1	F: TTTCTTCCTCAAAGGGACAT	CTAT_(15)_	54
		R: TTTGTATAAGGGCACAGGAA		
Amac-11	AOUJ01070479.1	F: CAGCAGGAGAATTTAAGCAA	GATG_(15)_	54
		R: CACTTTGTTGGAGGTGGTAA		
Amac-12	AOUJ01191554.1	F: GCAGTGCTCAGAAAGTAAGC	ATCT_(14)_	54
		R: TTCCTTCCCTCTGATATGGT		
Amac-13	AOUJ01191555.1	F: GCTTCAGTTGGTCATCAAAG	ATCT_(14)_	54
		R: AGCTGCAAATTAGGGAACTT		
Amac-14	AOUJ01209359.1	F: AAGTTGGAAGAAGACAGGATG	GGAT_(14)_	54
		R: GCAGCCACATAGAGCAATAA		
Amac-15	AOUJ01317281.1	F: AGCAGGACAGTAAAGGAAGG	GATG_(14)_	56
		R: AGGAATCAGCTCCAGACTTC		
Amac-16	AOUJ01434917.1	F: CCACAAAGGAAAACTCAATG	ATCT_(14)_	54
		R: CAGGGCTGTTATGAATGCTA		
Amac-17	AOUJ01122363.1	F: TTAGAGTTGCAGAGCAGGAC	ATCC_(14)_	54
		R: CAGGTCTCAGAACCCTTCTT		
Amac-18	AOUJ01095325.1	F: GAGCTCAGAGTGTGGACAAC	TGGA_(14)_	54
		R: GCAGTTCAGGCAATTAACAC		
Amac-19	AOUJ01194686.1	F: CATCATTTCCCTCTCTTCCT	ATAG_(14)_	54
		R: ACATGAGTACAGCGTCCATC		
Amac-20	AOUJ01457062.1	F: CACCCACCCAACAGTTAAT	GATA_(14)_	56
		R: TGCCTTTATAGACCCTTTCC		
Amac-21	AOUJ01152451.1	F: AGCAAAACCACATTCACATC	GATG_(14)_	54
		R: AAGTGGAGACCCTGACTGAT		
Amac-22	AOUJ01126529.1	F: CTCAGCTGACAGAGAGGAAA	TCCA_(14)_	54
		R: TCCAACAGAAGGCTTACAAA		
Amac-23	AOUJ01100929.1	F: GCACAGAGTGAGAAAGCAAG	ATCC_(13)_	56
		R: TAGTGTGGGGAACTCAAATG		
Amac-24	AOUJ01244951.1	F: GCAGAAGGCAAATAGGTTTT	GATA_(13)_	*na*
		R: GCAGAAGGCAAATAGGTTTT		
Amac-25	AOUJ01100098.1	F: ATTTCCGCTTAGGGTTAATG	CCAT_(13)_	54
		R: GTGAACATGAGGGAGACAAA		
Amac-26	AOUJ01052118.1	F: TAGCTTCGTGCTCTGCTAGT	TGGA_(13)_	54
		R: TCCTCCTACTTTGCTTCCTT		
Amac-27	AOUJ01155463.1	F: CTACTGTTCACCCAGAGCAG	ACT_(17)_	54
		R: TTGTGCTTTCCTACCTCTTG		
Amac-28	AOUJ01181657.1	F: TCATGTGTTCAAAACCTTCC	GCA_(17)_	*na*
		R: TAAACTCCAGAGCCAATGTG		

*na*: not amplified

The number of alleles and observed and expected heterozygosity of each locus were estimated using FSTAT (Goudet, 1995) and IDENTITY 4 (Wagner and Sefc, 1999). The linkage disequilibrium and deviations from Hardy-Weinberg equilibrium (HWE) were tested using GENEPOP 4.3 (Rousset, 2008), and its significance was assessed based on 10,000 permutations with 10,000 batches and 5,000 iterations per batch. For all analysis, *p*-values were adjusted for multiple testing controlling for false discovery rate (Benjamini and Hochberg, 1995). We checked for genotyping errors using MICRO-CHECKER 2.2.3 (Van Oosterhot *et al.*, 2004) with 1,000 Monte Carlo iterations and the Bonferroni confidence interval. Frequencies of null alleles were estimated in MICRO-CHECKER. Paternity exclusion probability and the probability of identity were estimated using IDENTITY.

A total of 1,706 potentially amplifiable microsatellite loci were found: 280 tri-, 999 tetra-, 330 penta-, and 97 hexanucleotides. The most frequent motif was AAAC (n = 228), followed by ATCC (n = 219) and AGAT (n = 145). In order to obtain a set of microsatellites of different motif lengths, we selected approximately 80% of tetra-nucleotide motifs and about 10% of penta- and trinucleotides. For each motif type, the loci with the highest number of repetitions (>10 repetition units) and the most heterogeneous in base composition were selected. Of the 28 tested loci, 26 showed amplification success, but three of them showed non-regularity in the amplifications (Amac-23, Amac-25, and Amac-27), and two did not generate amplification products (Amac-24 and Amac-28; see Tables 1 and 2). Annealing temperatures ranged from 54 to 56 °C (Table 1), and the sequencing results indicated homology between the fragments sequenced for *A. ararauna* and *A. macao*.

**Table 2:** Genetic diversity of 26 novel microsatellite loci for the Blue-and-yellow Macaw (*Ara ararauna*). Sample size, number of alleles (NA), size range (Min–Max), observed heterozygosity (H_O_), expected heterozygosity (H_E_), frequency of null alleles, probability of exclusion of paternity (PEP), and probability of identity (PI) are given for each locus.

Locus	Sample	NA	Min–Max	H_O_	H_E_	Null	PEP	PI
**Amac-01**	24	8	249–324	0.875	0.754	*ns*	0.536	0.098
Amac-02	21	8	298–396	0.476*	0.833	0.1948	0.668	0.049
**Amac-03**	23	11	294–349	0.870	0.858	*ns*	0.715	0.036
**Amac-04**	24	13	266–422	0.750	0.766	*ns*	0.561	0.088
Amac-05	23	13	300–448	0.826*	0.859	*ns*	0.735	0.031
**Amac-06**	22	6	231–251	0.818	0.777	*ns*	0.564	0.085
**Amac-07**	22	7	347–371	0.818	0.771	*ns*	0.566	0.085
**Amac-08**	23	9	336–376	0.826	0.850	*ns*	0.703	0.039
**Amac-09**	24	14	365–417	0.917	0.887	*ns*	0.774	0.023
**Amac-10**	24	10	274–330	0.792	0.720	*ns*	0.527	0.104
Amac-11	24	7	293–329	0.583*	0.779	0.1098	0.582	0.079
**Amac-12**	24	8	291–327	0.917	0.840	*ns*	0.679	0.046
**Amac-13**	23	15	363–415	0.826	0.890	*ns*	0.779	0.022
Amac-14	22	11	350–402	0.333*	0.833	0.2727	0.675	0.047
**Amac-15**	22	7	297–321	0.818	0.743	*ns*	0.540	0.096
Amac-16	21	6	337–357	0.381*	0.638	0.1571	0.415	0.170
**Amac-17**	24	6	223–243	0.833	0.720	*ns*	0.508	0.112
**Amac-18**	23	6	182–202	0.783	0.696	*ns*	0.457	0.141
**Amac-19**	24	6	213–233	0.833	0.766	*ns*	0.546	0.093
**Amac-20**	24	6	298–318	0.667	0.745	*ns*	0.523	0.105
**Amac-21**	24	4	380–416	0.417	0.353	*ns*	0.189	0.443
**Amac-22**	23	6	331–355	0.826	0.774	*ns*	0.561	0.086
Amac-23	4	5	385–413	0.750	0.688	*ns*	0.470	0.132
Amac-25	4	1	297					
**Amac-26**	24	9	206–254	0.667	0.811	*ns*	0.646	0.055
Amac-27	8	5	337–358	0.875	0.758	*ns*	0.529	0.100
*Total*							> 0.999	6.4e-21

* Departure from Hardy-Weinberg Equilibrium after false discovery rate correction; scoring error due to stuttering; *ns*: not significant; Loci names in bold indicate the set used to estimate the total PEP and PI.

From the 26 loci with amplification success, 25 were polymorphic and only one (Amac-25) was considered monomorphic. However, the latter was one of the loci that showed no regularity in the amplifications, and this state was based on the genotypes of four individuals. The number of alleles per polymorphic locus ranged from four (Amac-21) to 15 (Amac-13), with an average of 8.24 alleles per locus (Table 2). The observed heterozygosity ranged from 0.333 (Amac-14) to 0.917 (Amac-09 and Amac-12) and the expected heterozygosity ranged between 0.353 (Amac-21) and 0.890 (Amac-13; see Table 2). Linkage disequilibrium was not found for any pairwise comparison. Most loci were in HWE, except for five (Amac-02, Amac-05, Amac-11, Amac-14, and Amac-16; see Table 2). According to the MICRO-CHECKER results there is significant evidence for a null allele at Amac-02, Amac-11, Amac-14, and Amac-16 loci, and evidence for stutter peaks at locus Amac-16. Based on the set of 18 polymorphic loci with high amplification success and under HWE (see Table 2), we found high paternity exclusion probability (> 0.999) and low probability of identity (6.40e^-021^).

Six dinucleotide microsatellites loci previously isolated for *A. ararauna* and genotyped for 49 individuals showed number of alleles varying from one to 11 per locus (Caparroz *et al.*, 2003), being less polymorphic than the set presented here. Additionally, those loci were composed by dinucleotide motifs, which are sometimes difficult to score due to the presence of stutter bands. In addition to being composed by tri-, tetra-, and pentanucleotides, the loci presented here also showed high level of polymorphism, indicating that this set will be useful in the analysis of genetic diversity and population structure of *A. ararauna* and potentially other macaw species. The high paternity exclusion probability and low probability of identity indicate the potential for the use of these loci in parentage analyses of *A. ararauna*, helping authorities to check and control commercial breeders. Moreover, these new microsatellites will help studies about the social structure and mating system of this and other related species.
